# Angiosarcoma Mimicking Rhinophyma

**DOI:** 10.1155/2010/365173

**Published:** 2010-06-06

**Authors:** Maurizio Lo Presti, Caterina Mazzella, Ambra Monfrecola, Jessica Falleti

**Affiliations:** ^1^Department of Pathology Systematics, Section of Dermatology, University Federico II of Naples, via S. Pansini 5, 80131 Naples, Italy; ^2^Department of Biomorphological and Functional Science, Section of Pathology, University Federico II of Naples, 80131 Naples, Italy

## Abstract

We report the case of a 61-year-old man showing persistent erythematous macules, plaques, and partially confluent nodules with irregular borders, developed on his nose for one year. During that time the patient underwent several dermatological consultations, and all produced the same diagnosis: rhinophyma. So antibiotic and steroid treatment was carried out without any improvement while the lesions kept growing. When the patient came to our observation, physical examination revealed large, infiltrative, oedematous, erythematous plaques and rare nodules, with superficial telangiectatic vessels. Cervical lymphadenopathy was not detectable. Routine laboratory analysis was normal. Punch biopsy was performed, and histopathology and immunohistochemical studies were consistent with cutaneous angiosarcoma. This is the report of a face angiosarcoma with an unusual and very deceptive clinical presentation.

## 1. Introduction

Angiosarcoma (AS) is a rare malignant vascular tumour that arises in skin or superficial soft tissue in 60% of cases [1]. It mainly appears on the head and neck of elderly patients. Clinical manifestations of AS are characterized by erythematous plaques, macules, or nodules and sometimes by chronic edema of the face. Diagnosis may often be delayed by the variable presentation and the benign appearance of the lesion, therefore, histological features are fundamental for a correct diagnosis.

 The prognosis is poor because the tumour is aggressive with high local recurrence and early metastasis.

## 2. Case Report

**Figure 1:**
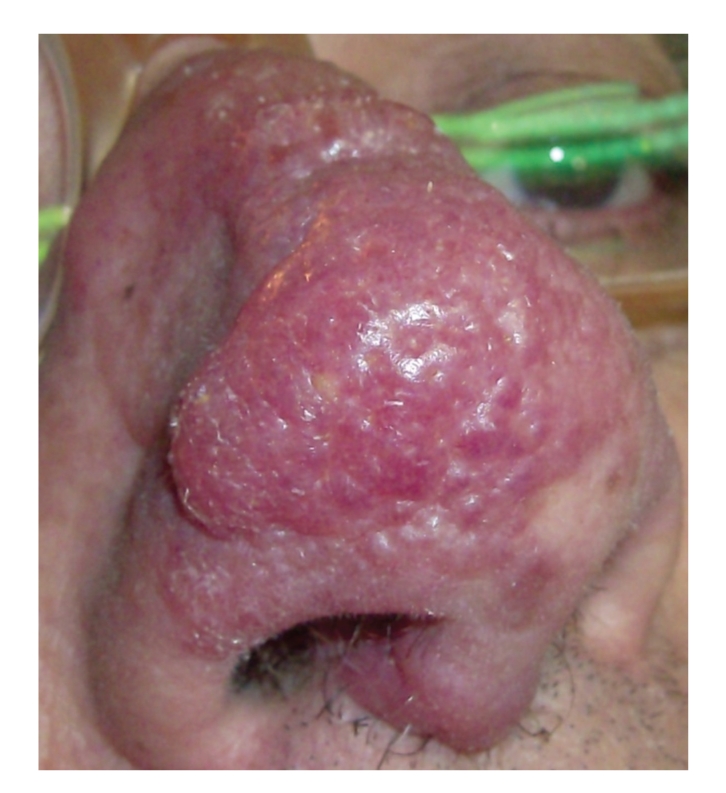
Close up view Erythematous macules, plaques, and rare nodules with irregular borders on the nose with superficial teleangiectatic vessels.

A 61-year-old man came to our department with erythematous macules, plaques and also rare nodules with irregular borders, localized on the nose, developed for about one year (Figure 1).

Before examination at our clinic, the patient was repeatedly treated for rosacea/rhinophyma with oral tetracyclines, metronidazole, and steroids without significant improvement. 

He also suffered of hypertension, ischemic heart disease, and benign prostatic hyperplasia.

**Figure 2:**
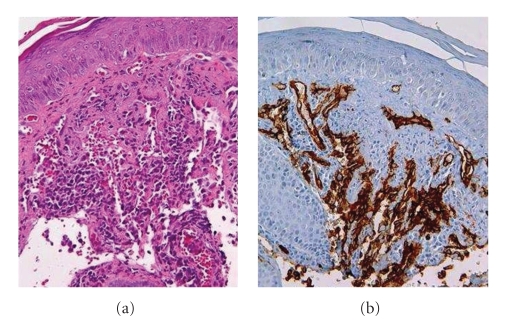
(a) Neoplastic vascular proliferation lined by atypical and plump endothelial cells in the dermis. (b) Positivity of neoplastic endothelial cells for CD 34.

Histopathologic examination of skin biopsy, taken from a nodule, showed vascular proliferation lined by atypical and plump endothelial cells in the dermis; immunohistochemistry proved the positivity of neoplastic endothelial cells for CD31 and CD34 (Figures 2(a), 2(b)).

 Based on the clinical and histopathological findings, diagnosis of angiosarcoma of the nose was established. Total body CT, showing no significant findings, was performed, and then, according to consolidated cooperation, and also following his own desire, the patient has been put under oncologists care, undertaking Rx therapy (electron beam).

## 3. Discussion

Angiosarcoma (AS) is rare, amounting for less than 2% of all sarcomas. Skin and superficial soft tissue are the most common locations (60% of cases) [2] with high rate of lymph node and systemic metastasis and reported 5-year survival of 10% to 35% [3]. 

AS of the skin is often classified as

- AS on the face and scalp of elderly population,
- AS either in association with chronic lymphedema or secondary to a prior surgery (Stewart-Treves syndrome),
- AS following chronic radiodermatitis or skin trauma and ulceration [4].

Our case can be described as AS of the face, that, together with scalp AS, is the most frequent type, with a poor prognosis and a 5-year survival rate of 15% [3].

The etiology of AS remains unknow, but, as many cases arise in the elderly and many patients have a long history of solar and/or chronic ultraviolet exposure, solar damage has been supposed but not confirmed as pathogenetic factor. Few cases have been correlated to other factors as radiation, vinyl chloride, arsenic, insecticides, androgenic steroids, and thorium dioxide exposure. Actually there are no known genetic defects or chromosomal abnormalities implicated in AS pathogenesis [5].

The classical manifestation is characterized by erythematous plaque which expands rapidly involving large areas of the skin, forming blue-black nodules that often ulcerate. Facial oedema can develop too [6]. Sometimes AS may appear as cellulitis or erysipelas [7] seldom and as xanthelasma or scarring alopecia [1]. 

The tumour often mimics many different conditions such as arteriovenous malformations, nodular melanoma, lymphoma, sarcoidosis, or facial granuloma [3]. 

The clinical picture described in this paper strongly mimicked rhinophyma for a long period of time, to the point that it was repeatedly treated by steroids and oral antibiotics, further delaying the correct diagnosis.

In the literature both AS developing on rhinophyma and AS resembling rhinophyma are reported. The former was described by Gallardo et al. (2000) [6] and the latter by Aguila and Sánchez (2003) [3] who also showed, by means of histopathological examination, a lymphocytic infiltrate in the lesions, similar to our case. 

Some authors believe that nasal AS has a better prognosis due to the lower grade nature and to a shorter interval between diagnosis and treatment, but there are few reports accurately describing the survival periods, moreover, different survival rate can be related to differences in treatment [7] and even to tumour's size [8].

Therapeutical approach comprises wide resection in localised neoplasms while for larger tumors radiotherapy or chemotherapy can be used, even if no standard therapeutic protocols have been yet established [9].

The present case is reported because of its peculiar and rare clinical presentation, characterized by marked rhinophyma-like features making it a difficult but intriguing diagnostic challenge.
